# Male mice adjust courtship behavior in response to female multimodal signals

**DOI:** 10.1371/journal.pone.0229302

**Published:** 2020-04-02

**Authors:** Kelly L. Ronald, Xinzhu Zhang, Matthew V. Morrison, Ryan Miller, Laura M. Hurley

**Affiliations:** 1 Department of Biology, Indiana University, Bloomington, IN, United States of America; 2 Department of Biology, Hope College, Holland, MI, United States of America; 3 Department of Evolution, Ecology, and Organismal Biology, The Ohio State University, Columbus, OH, United States of America; Duke University, UNITED STATES

## Abstract

Multimodal signaling is nearly ubiquitous across animal taxa. While much research has focused on male signal production contributing to female mate-choice or preferences, females often give their own multimodal signals during intersexual communication events. Multimodal signal components are often classified based on whether they contain redundant information (e.g., the backup hypothesis) or non-redundant information (e.g., the multiple messages hypothesis) from the perspective of the receiver. We investigated the role of two different female vocalizations produced by the female house mouse (*Mus musculus*): the broadband, relatively low-frequency squeaks (broadband vocalizations or BBVs,), and the higher-frequency ultrasonic vocalizations (USVs). These female vocalizations may convey differently valenced information to the male receivers. We paired these vocalizations with and without female urine to examine the influence of combining information across multiple modalities. We found evidence that female urine and vocalizations act as non-redundant multimodal cues as males responded with different behaviors and vocalization rates depending on the female signal presented. Additionally, male mice responded with greater courtship effort to the multimodal combination of female USVs paired with female urine than any other signal combination. These results suggest that the olfactory information contained in female urine provides the context by which males can then evaluate potentially ambiguous female vocalizations.

## Introduction

Studies of intersexual communication often examine courtship signaling from the perspective of the ‘showy' male being the signaler and the ‘choosy' female being the receiver [1]. As such, the intersexual communication literature is biased such that we know much about the production of male signals and how females respond to them, and relatively less about female signal production. Consequently, we know little about whether male courtship behavior is affected by the reception of female signals. Even in scenarios where female signaling is recognized, like during duetting behavior, the function of those signals or the information they contain generally remains unknown [2–4]. One of the few notable exceptions is in the brown-headed cowbird (*Molothrus ater*) where adult females can change the development of a younger male’s courtship song with a fast, visual wing-flick; females will give this wing-flick if they prefer the song elements the male is producing, and the males subsequently modify their songs to be more aligned with the female preferences [5]. This example illustrates that during intersexual communication, female signals can ultimately shape the design and use of male courtship signals. Ultimately, we expect courtship communication to be a dynamic exchange of information between the sender and receiver where both males and females alike are producing and receiving signals.

Moreover, signals used during communication events are often complex and can combine information across sensory modalities (e.g., multimodal communication) [6–8]. While the use of multimodal signals by males during courtship has now been described across a wide range of taxa (for reviews see [6, 7], we are generally less aware of whether males attend to female multimodal signals. Multimodal signals have typically been classified in terms of the information each signaling component contains with the use of cue-isolation experiment s(i.e., the content hypotheses). Such experiments evaluate the behavior of the receiver when it is presented with a signal component in isolation. If the receiver responds in a similar way to each signaling component it is assumed that the information contained in each modality is the same and therefore each modality serves as a "back-up" to one another (i.e., the redundant signaling hypothesis, [9]. In comparison, the response to the full multimodal signal is predicted to be either equivalent to either signal component presented alone, or enhanced [9]. This redundancy in signal components is expected to benefit the receiver especially in uncertain environments where some signal components may not reach the receiver. In contrast to responding similarly to redundant signal components, receivers respond in different ways to non-redundant signal components [9] and therefore it is assumed that each component provides different information to the receiver (i.e., the non-redundant signaling hypothesis, or "multiple messages" hypothesis, [9]. The response to the full multimodal signal may then be an independent response to both signal components (i.e., independence); alternatively, the response could be an interaction of these two modes such that one component is dominant to the other (i.e., dominance), one may modulate the response to the other (i.e., modulation), or an entirely new behavior could emerge (i.e., emergence). Non-redundant signal components benefit the receiver by providing two measures of quality or a measure of quality and extra information like species identity or location [6].

The house mouse (*Mus musculus*) is a highly social species [10, 11] that uses both acoustic [12–15] and chemical [16–20] signals to communicate with conspecifics. For example, males produce ultrasonic vocalizations (USVs) that may function as courtship signals to females [21–24]. Male USVs are highly variable between species [25] and individuals [15, 26, 27] and may therefore convey individual identity [28]. Females, in turn, prefer the USVs from novel males [15] within their own species [25]. Interestingly, female preference for male USVs also varies with previous exposure to male odors (e.g., soiled bedding) and female estrus state [27]. Female mice are particularly attracted to male urine with a high level of the protein, darcin [29]. Interestingly, both the number of male USVs and the amount of darcin in urine seem to be influenced by male immune system functioning [30]. Together, these results suggest that female preferences for male signals cannot just be ascribed to their attraction to male USVs in isolation. Rather, both USVs and olfactory signals interact to influence female preferences. How and whether males respond to female multimodal signals, however, remains relatively unknown.

Originally it was thought that female mice rarely produce USVs during intersexual encounters [31]; however, new evidence suggests that females produce about 15% of the total USVs during these communication events [32]. Differences between male and female USVs have been historically challenging to study because they are structurally very similar [33] and require extensive microphone arrays in order to distinguish which mouse vocalized [32]. Nevertheless, it was recently found that the females produce USVs in temporal synchrony with male USV production, and often when males are in pursuit (i.e., chasing) of females [32]. Females that produce USVs during these chases are effectively slower than silent females. Together, these results suggest that female USVs may indicate increased female receptivity to male courtship efforts [32].

Female mice also produce lower-frequency, broadband vocalizations (BBVs) that are commonly referred to as ‘squeaks’ [13, 34, 35]. Female BBVs are also produced during intersexual interactions, including when males are exhibiting mounting behaviors [13]. In contrast to USVs, however, squeaks produced early during a male-female interaction are thought to be an indication of female rejection behavior because (1) they are highly correlated with other rejection behaviors like kicking and lunging and (2) female squeaks earlier in intersexual interactions correspond to less mounting attempts by the male [13]. Therefore, female mice may use these two different vocalizations, BBVs and USVs, to communicate their level of receptivity to males during the early stages of an interaction. This information could in turn alter the level of male courtship effort and ultimately, whether an interaction leads to copulation.

Males also attend to other information from sensory modalities like chemical cues in addition to female vocalizations. It is well established that males will produce more USVs to female urine than male urine [18]. In fact, males with experience with the opposite sex will even produce USVs to soiled female bedding without the presence of a live female [31]. Numerous studies have investigated the factors influencing male USV responses to female urine or olfactory cues. Males produce significantly more USVs when presented with urine from sexually mature females compared to immature females [17]. The spectral and temporal nature of male vocalizations differ with female estrous phase [12] and male mounting behavior is related to the presence of female estrogen sulfates in urine [36]. Furthermore, males will produce fewer USVs to ovariectomized females rather than intact females [37]. Furthermore, males produce more USVs to novel female urine than to familiar female urine [15]. These studies indicate that urine contains information regarding a female’s age and possible reproductive receptivity and identity.

Female vocalizations and odor cues can function in different temporal and spatial scales: while deposited urine can transfer information over a relatively longer time frame and spatial scale, mouse vocalizations are short in duration and high in frequency and therefore propagate over short distances ([38, 39]. This time and space difference paired with the research described above suggests that these two signaling components may contain different information for the male receiver (i.e., the multiple messages hypothesis). From this hypothesis we predicted that males should respond differentially to each signaling component (e.g., urine, USVs, squeaks) when they are presented in isolation. Our experimental design included a period of time prior to the stimulus playback to which we could then compare any behavioral changes after stimulus presentation. Therefore, we were highly interested in the presence of a statistical interaction between our stimulus presentation and the presentation time in our analyses. Such an interaction would indicate that males changed their behaviors depending on both the presentation time and the type of stimulus presented.

Specifically, we predicted that (1) males should respond with more investigative behavior and USV production to female USVs than to other unimodal stimuli as USVs may indicate the presence of a nearby, receptive female and (2) males will show *decreased* investigation and USV production when presented with female squeaks as squeaks may convey female rejection. In comparison to the unimodal presentation, we expected that the multimodal combination would be more salient to the male listener and that the two modalities would interact to modulate the behavioral responses [9]. Therefore, we also predicted that the (3) USV and urine combination would elicit the highest degree of USV production and investigation and (4) that the squeak and urine combination would show the lowest degree of USV production and investigation.

## Methods

### Animals

Focal subjects (N = 9) were male CBA/J mice (the Jackson Laboratory, Bar Harbor, ME, U.S.A) aged 9–10 weeks. Mice of this strain are bred to have better hearing than many lab strains (Zheng, Johnson et al. 1999) An additional 24 non-virgin females and 6 non-virgin males were used as stimulus animals for recording female vocalizations, collecting female urine, or for giving opposite-sex social experience. All focal animals were housed in same-sex social groups of 3 mice in standard plastic cages for laboratory mice (28.5 x 17.5 cm and 12.5 cm tall) with pine bedding and nesting material. Females used for urine collection and vocal recordings were housed in pairs or groups of 3. These females may have had outside exposure to males (outside of the focal males included in this study) during the period of time urine collection occurred in other experimental protocols where female conspecifics were needed as stimuli. For example, some females may have been used to give other males (i.e., not included in this study) sexual mounting experience. Nevertheless, no females in our study became pregnant during the urine collection period and therefore we are confident that the pooled urine in our experimental did not include urine from pregnant individuals. We cannot, however, rule out the possibility that some females may have been in a phase of prolonged diestrus as a result of the Lee-Boot effect [40, 41]. Nevertheless, data from our lab shows that females housed in groups of 2 or 3 with other females typically go through their estrous cycle within 4 days [12, 13, 42, 43]; (also see S1 File) which is a normal length of time [44]. This suggests that the Lee-Boot effect may be minimal in our study, perhaps because of the exposure to male mice or because of the relatively small group size [44]. All animals were provided with ad libitum food and water and housed on a 14:10 light:dark cycle. One week prior to behavioral tests, focal males were paired with an unfamiliar stimulus female (not used for vocal recordings or urine collection) so that males could gain experience with the opposite sex. Focal males interacted with three different female partners 3 times in 10 min interactions over 3 consecutive days (i.e., 9 interactions); all males mounted their female partner at least once during one of these interaction periods.

### Ethical note

All animal use procedures were approved by the Indiana University, Bloomington Institutional Animal Care and Use Committee (protocol 18–025). Care was taken to ensure compliance with animal welfare guidelines to minimize the welfare impact on subjects. All mice were housed in social groups and provided nesting material for environmental enrichment to reduce stress with laboratory housing. Animals were habituated to being handled when they were provided with opposite-sex social experience in pairs. Furthermore, for two consecutive days prior to the experiment, focal males (N = 9) were placed into the experimental arena for 30 minutes to habituate them to the testing arena. In addition, our repeated measures design allowed us to reduce the overall number of animals used in this experiment.

### Urine collection

We collected urine from 18 non-virgin female CBA/J mice twice daily over the course of February-April 2017 to generate a pool of urine from which we could aliquot into experimental amounts. Female mice go through estrus on average every 4 to 5 days [45] and previous work from our lab [12, 13, 42, 43], as well as new data from our lab (see S1 File) has assessed female estrous state across this normal range of rodent cycling. Interestingly, male mice mount females regardless of their estrous state [12], and produce USVs and all USV syllables at the same rate to females whether they are in estrus or diestrus [12]. Therefore, although we did not examine females for estrous state in this study, it is likely that daily sampling over two months included sampling from females that were in estrus and diestrus. Moreover, no females were actively pregnant or became pregnant during this study so the urine pool only contained urine sampled from non-pregnant individuals. Additionally, urine was collected from 6 males to serve as a stimulus for recording female USVs.

Urine was collected over a clean sheet of aluminum foil by handling the mouse and immediately pipetted into a centrifuge tube on dry ice. Because we were uncertain whether there would be a response to multimodal stimuli we opted for a conservative design of pooling all urine (within a sex) with the understanding that our findings would not be selective for estrous phase, female identity or kinship. Urine was stored within centrifuge tubes in in an -80 freezer until enough urine was collected for the entire behavioral experiment. In May 2017, all frozen urine was thawed, pooled together within a sex, and portioned in 60 μL aliquots. Urine was then refrozen at -80°C until needed immediately before an experimental trial.

### Behavioral setup

**Fig 1:**
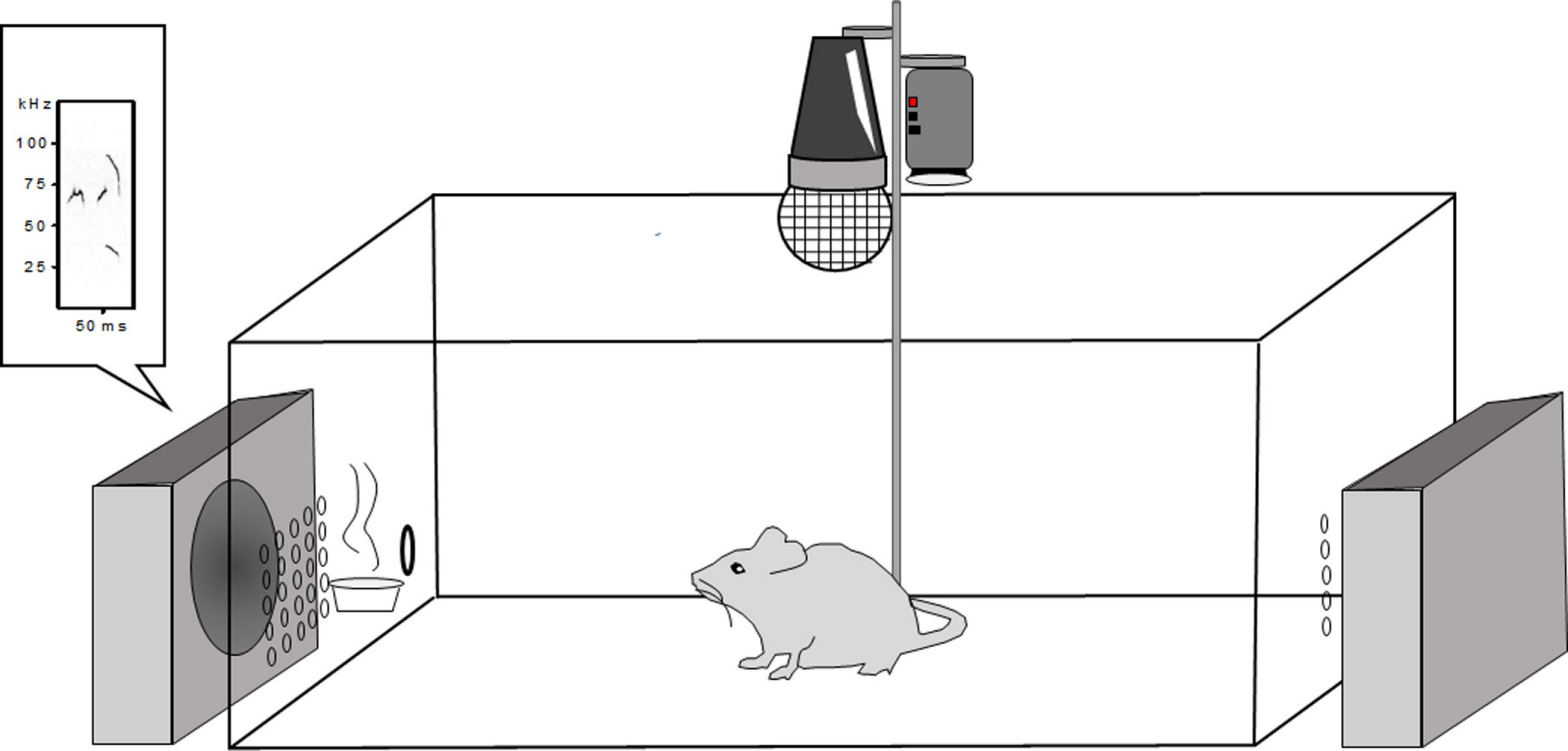
Schematic illustration of the experimental arena. A simple figure of the experimental arena for the recording of all focal animals. A speaker and an investigation hole are on opposite sides of the cage so that stimuli could presented randomly at each side of the cage. Urine is presented on filter paper outside of the investigation hole. An USV-sensitive microphone and video camera record the focal animal’s vocalizations and behaviors, respectively.

All behavioral and vocal recordings took place in an acoustic isolation chamber (Controlled Acoustical Environments, Industrial Acoustics Company, Inc.) fitted with a light source and a camera (Canon Vixia HFR700) attached to a ring stand for top-down viewing of the experimental arena (see Fig 1). All vocalizations were recorded via an Avisoft-UltrasoundGate 116H Recorder (#41163; Avisoft Bioacoustics, Berlin, Germany with a sampling rate of 250 kHz) attached to a Dell Optiplex 960 Computer running Avisoft Recorder Software and a 16-bit condenser microphone (CM16/CMPA; Avisoft Bioacoustics, Berlin, Germany; 200 kHz maximum range) directly above the arena. The camera was attached to a Sceptre Model E22 Monitor for real-time viewing on the outside of the isolation chamber.

### Female vocalization recordings

Six non-virgin CBA/J females were used for recording both female squeak and female USV stimuli. Female squeaks were recorded from all 6 females by placing a male and a female together into a standard mouse cage with clean bedding fitted within the behavioral setup arena for 20 minutes. Females often squeak early during an encounter with a male as he begins to attempt mounting [13]. Female USVs were recorded by placing 60 μL of previously frozen male urine on a cotton ball in the center of a standard cage and allowing a single female to explore the arena for 20 minutes. Placing a female in an isolated cage for USV recordings rather than with a vocally-interacting male ensured that we only collected female-produced USVs for our playback study. All recordings were saved as separate .wav files and spectrograms were generated in Avisoft SASLab Pro software (Avisoft Bioacoustics) with an FFT length of 512 and a Hamming style window with 50% overlap.

### Female vocalization playback design

New stimulus spectrograms for playback (i.e., one squeak file and one USV file) were generated pseudo-randomly (i.e., randomly provided that the vocalization was not obstructed by noise) by selecting recorded female squeaks or USVs, respectively, from across the 6 females. The squeak file replicated the natural progression of female squeaks during interaction with a male across 1-minute bins previously reported in [13]. We found that female squeak production generally followed a bell-curve distribution and therefore we modelled our squeak playback similarly; over the course of 5 minutes, 10 squeaks were evenly distributed over the first minute, 20 squeaks in the second minute, 40 squeaks in the third, 20 squeaks in the fourth minute, and 10 squeaks in the final minute. Thus, the final playback had a total of 105 unique squeaks recorded from 6 different females. We designed the USV file in a similar way: we created a five-minute sequence that had 6 USVs distributed evenly over the first minute, 12 in the second minute, 24 in the third minute, 12 in the fourth minute, and 6 in the final minute. This resulted in a playback of 60 total USVs produced over the course of 5 minutes. To calibrate the loudness of our sound file during play back, the same microphone was used to record the playback of our recording from a speaker (#60108; Avisoft Bioacoustics, Berlin, Germany; Frequency range ±12dB: 1–120 kHz) placed on the side of the arena. Loudness of the USVs were measured by RMS amplitude on a spectrogram using Avisoft Software and compared to the RMS amplitude of the original file. Speaker intensity level was chosen on which intensity level resulted in an RMS closest to the original file.

### Behavioral arena

The testing arena was made from a standard mouse enclosure fitted with a grid of small holes centered 1 cm apart, in rows of 12 x 14 on both short sides of the cage to allow propagation of vocal signals. Next to the grids, were a hole 7/16 of an inch in diameter at opposite ends of the cage to serve as ‘investigation circles’ (see Fig 1). Outside of the investigation holes we placed a small weigh boat with a circular Whatman filter paper (42.5 mm).; This weigh boat is where 60 μL urine (thawed immediately prior to presentation) could be presented to the mouse through PTFE tubing (Component Supply Company, SWTT-30-C) attached to a 30.5-gauge needle and syringe outside of the testing chamber. Two Ultrasonic Dynamic Speakers (#60108; Avisoft Bioacoustics, Berlin, Germany; Frequency range ±12dB: 1–120 kHz) were placed on opposite ends of the cage, next to the investigation holes. A cup of clean pine bedding as added to the arena right before an experimental trial; in between trials the bedding was discarded and the arena was cleaned with 70% ethanol.

### Behavioral experiments

Behavioral experiments were run from 9 a.m. till 3 p.m. during the light portion of the light:dark cycle with each male exposed to one treatment per day over the course of 10 days. We used a complete and balanced repeated measures design in which each male was exposed to every condition with repetition. Each male was randomly exposed to each of the five treatments twice (e.g., USVs only, squeaks only, urine only, urine+squeaks, urine+USVs). This resulted in 90 total trials across all the focal males (see S2 File for a file outlining our experimental protocol). Importantly, previous work in our lab has demonstrated that there is a fairly high degree of individual variation in male USV production [12]. For these reasons, it was important to place all males in all conditions in order to be able to strongly conclude that the stimulus, rather than male identity, was the driving force between any differences in response.

This repeated measures design allowed us to distinguish whether the differences between treatment types was larger than the variation within males.

A behavioral trial began when a randomly selected male was placed into the experimental arena for 10 mins of habituation time while video and audio recording occurred to serve as a baseline for vocal and non-vocal behaviors. After ten minutes, a light switch was briefly flipped (which also produced a low-intensity noise) to indicate in the video and audio recording when the treatment was being presented. The stimuli were presented at a random chosen side of the arena (to control for side bias) and recordings continued for an additional 15 minutes. After a trial, males were weighed and returned to their home cages.

### Behavioral and vocal analysis

We analyzed five different nonvocal behaviors (i.e., investigation of the stimulus and non-stimulus circle, rearing, digging, and grooming) from our video recordings using ODLog software (Macropod Software, http://www.macropodsoftware.com/). *Investigative behavior* was defined as males placing their noses through either of the “investigation circles” at which olfactory stimuli were presented on two opposite sides of the cage. We coded each of these circles differently so that we could differentiate investigation at of the stimulus and the exploration of the other non-stimulus circle. *Rearing* was defined as mice standing on their hind legs while their fore legs were against the side of the cage. Rearing is often taken as an indication of relaxed exploratory behavior in mice [46]. *Digging* was defined as mice moving bedding substrate with both fore and hind legs. In mice, digging is a natural behavior related to burrowing or exploration, and is also used as a model of obsessive-compulsive-like behavior [47, 48]. In our past work, digging has increased in response to playback of vocalizations [43].*Grooming* was defined as mice using their fore legs or mouth to clean their own fur. Rodents may groom in response to olfactory signals, in response to conflicting behavioral impulses, or as an anxiety-like behavior [49]. All of these non-vocal behaviors were performed at relatively high rates.

Avisoft SASLab Pro software (Avisoft Bioacoustics) was used analyze all spectrogram recordings of USVs males produced during the trials. We high-pass filtered all audio files above 35 kHz to remove the majority of the background noise. We then used the interactive (section labels) feature in the automatic parameter measurements setup to manually select the USVs. In addition to quantifying the overall rate of USVs by counting the number of vocalizations per unit time, we also classified the USVs into two different types of vocalizations (1) vocalizations with a harmonic structure with a fundamental frequency at 50 kHz and (2) ‘Others’. Previous work has shown that 50 kHz harmonic vocalizations are important during intersexual interactions are especially given by males in high numbers around mounting behaviors [12, 13, 50, 51]. We also measured the total duration and the frequency at maximum intensity to examine if there were spectral or temporal differences in the types of USVs made with different stimulus conditions. Previous work in our lab has shown that these two vocal features are sensitive to the presence or absence of female stimuli [12].

### Statistical analyses

We examined how males changed in 1) the proportion of non-vocal behaviors they performed and 2) the number of USVs they produced, before and after the presentation of the stimulus. We used general linear mixed models (Proc GLMM) in SAS (version 9.3) with male identity as a repeated factor to investigate our predicted statistical interaction between the playback time (i.e., the period before versus after the stimulus was presented) and treatment stimulus. A significant interaction indicates that male mice changed their behavior after the stimuli presentation and that this change was dependent on which treatment was being presented. This approach of comparing behavioral changes before and after treatment presentation, within a repeated measures design, allowed us control for high variability between males so that we could distinguish differences due to treatment presentation.

We examined five different non-vocal behaviors including (i.e., investigation of both the stimulus circle and non-stimulus circle, rearing, grooming, and digging). In each analysis we separately modelled the behavior of interest as the dependent variable, and the main effects of stimulus treatment, playback time, and their interaction as independent factors. In addition, we also explored whether males changed the rate of the 50 kHz harmonic calls or the temporal or spectral structure of their USVs depending on the stimulus presented. Therefore, we ran 5 different models to investigate changes in non-vocal behaviors across our subjects, and 4 models to investigate vocal behaviors (i.e., total USV rate, 50 kHz harmonic USV rate, duration of the total USVs, and dominant frequency of the total USVS). All significant interactions were explored with the use of post-hoc t-tests. We applied the Benjamini-Hochberg correction to correct for multiple comparisons during our posthoc analyses [52].

In addition to our independent variables of interest we also included several covariates within our models. We included the trial day because previous studies have shown habituation to the testing arena [23], the mass of the male as this may be related to dominance status and USV production [53–56], and the playback position (to investigate any side bias). Our repeated measures design across several days also allowed us to control for these covariates in our statistical models. We log transformed the mass of the animal and used an arcsine transformation of the behavioral dependent variables to meet the normality assumption. We also included male and stimulus treatment within male as random factors in a random statement. In addition, we specified an autoregressive covariance structure, and the Kenward-Roger method was used to calculate the degrees of freedom. Post-hoc t-tests allowed exploration of any significant interaction effects. Least square (LS) means for creating figures (presented below) were generated from the ‘lsmeans’ statement in SAS; this statement computes the LS means of fixed effects and are predicted population margins[57]. One benefit to using LS means is that they take into account the structure of the statistical model when producing the mean value such that the means reflect the effects of any covariates (i.e., trial day and stimulus presentation side) included within the model[57]. Within this statement each each mean is calculated by the product of L($\hat{\beta }$). Here, L is the coefficient matrix associated with the least squares means and $\hat{\beta }$ is the estimate of the fixed-effects parameter-vector[57].

## Results

### Male non-vocal behaviors

**Fig 2:**
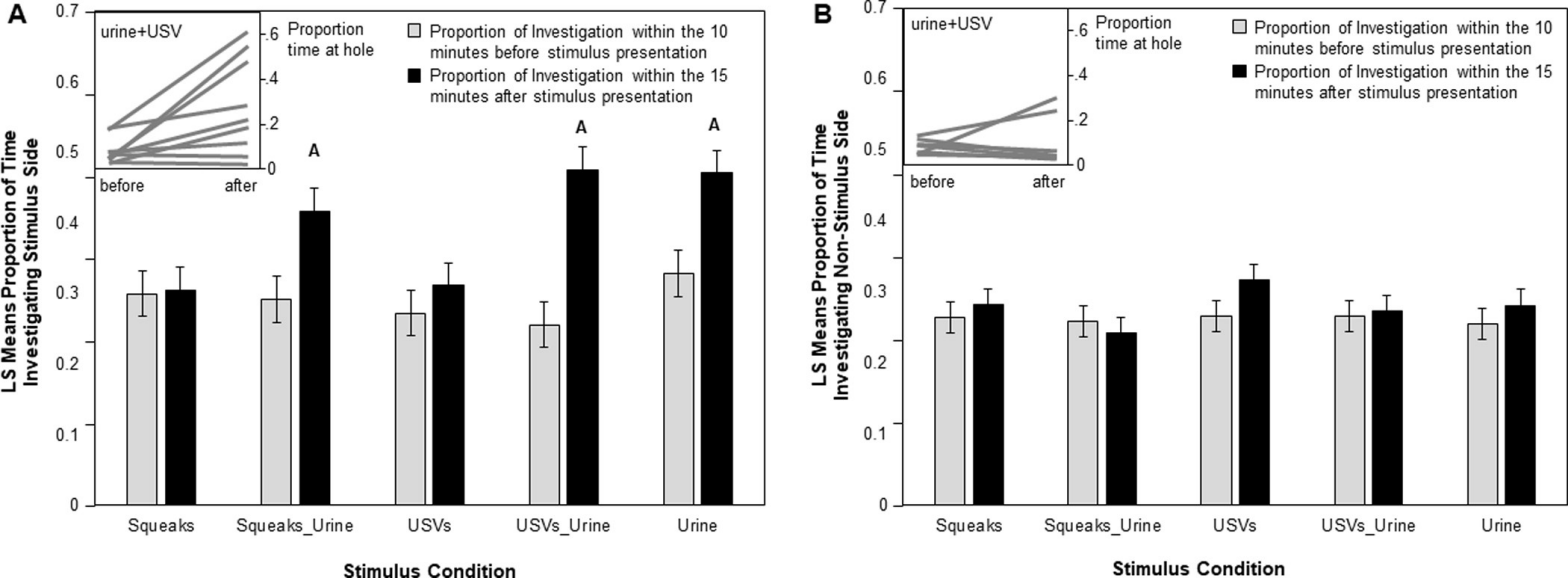
Male investigative behaviors are affected by stimulus condition. Panel A shows the Least Squares (LS) Means for the proportion of time spent investigating the side of the cage the stimulus was presented on. Panel B shows the LS Means for the proportion of time spent investigating the alternative side of the cage. Gray bars refer to before the stimulus was presented and black bars refer to after stimulus presentation. Letters designates a significant interaction term between stimulus type and presentation time; i.e., a significant increase in investigation from before stimulus presentation to after dependent on stimulus type. Insets depict proportional times spent at target windows for individual mice in the urine + USV condition during the 10 minutes before, and 15 minutes after stimulus presentation, respectively.

The non-vocal behavioral responses of males varied with the types of female signals that were presented. We found a significant statistical interaction between stimulus type and presentation time, indicating that mice changed their behavior after the stimulus presentation and as a function of which type of stimulus presentation. Specifically, males investigated more when female urine was present regardless of whether female urine was presented alone or paired with a female vocalization (Table 1). Indeed, males spent significantly more time investigating when female urine was presented compared to when only USVs were presented (t_65.7_ = -3.50, p = 0.002) or to when only squeaks were presented (t_65.3_ = -3.65, p = 0.002) (Table 1 and Fig 2A).

**Table 1:** Male investigative behaviors are affected by stimulus condition.

	Investigation of Stimulus Investigation Circle			Investigation of Non-Stimulus Investigation Circle		
	D.F.	F value	P value	D.F.	F value	P value
**Stimulus**	**4, 36.7**	**2.7**	**0.05**	4, 27.8	0.95	0.45
**Time (Before or After)**	**1, 58.8**	**55.99**	**< 0.001**	1, 56	2.14	0.15
Stimulus*Time	**4, 97.7**	**6.23**	**< 0.001**	4, 109	0.8	0.53
**Trial Day**	**1, 61.4**	**11.09**	**0.002**	**1, 63.7**	**4.32**	**0.04**
**Log(Mass)**	1, 63.7	0.71	0.4	1, 26.2	1.27	0.27
**Stimulus position**	**1, 117**	**43.8**	**< 0.001**	**1, 93.2**	**98.42**	**< 0.001**

Results of two models; first run using ‘investigation of the Stimulus-Investigation Circle’ as a dependent variable and second using ‘Investigation of Non-Stimulus Investigation Circle’ as a dependent variable. We see a significant interaction here between stimulus type and the time of the presentation (i.e., before versus after the stimulus was presented) on investigation of the stimulus circle. Stimulus refers to the type of female stimulus condition presented, time is related to before or after the stimulus presentation. Stimulus position refers to the side of the cage that the stimulus was presented on. Significant values are in bold.

Males did not spend more time investigating the multimodal stimuli (i.e., urine+USVs (t_65.1_ = .10, p = 0.92) or urine+squeaks (t_65.7_ = -1.17, p = 0.31) compared to female urine presented in isolation (Fig 2A). The increase in investigative behavior with urine presentation only occurred at the investigative circle where stimulus was presented, not at the alternative circle (Table 1 and Fig 2B), suggesting that the change in male behavior was in direct response to female signal presentation. In contrast, males did not change their investigative behavior with the presentation of just USVs (t_89.2_ = 0.04, p = 0.31) or just squeaks (t_86.1_ = .18, p = 0.92) (Fig 2A).

No other non-vocal behaviors tested, including self-grooming, rearing, or digging (Table 2) showed a significant interaction between stimulus presentation (e.g., before and after stimulus) and stimulus treatment. Moreover, we also did not find a significant main effect of stimulus on any of these behaviors. Therefore, we did not do any further posthoc analyses to investigate the interactions. There was, however, a significant main effect of the time of stimulus presentation on rearing and digging (Table 2). Overall, males decreased the proportion of time they spent rearing (before = 0.35 ± 0.008, after = 0.28 ± 0.008), and digging (before = .24 ± 0.001, after = .20 ± 0.001) after the stimulus presentation.

**Table 2:** Self grooming, rearing, and digging behaviors are not affected by any female stimuli.

	Self-Grooming			Rearing			Digging		
	D.F.	F value	P value	D.F.	F value	P value	D.F.	F value	P value
**Condition**	4, 30.4	1.15	0.35	4, 31.1	0.83	0.51	4, 22	1.16	0.36
**Time (Before or After)**	1, 65.7	0.02	0.88	**1, 58.1**	**90.73**	**< 0.001**	**1, 85.5**	**27.23**	**< 0.001**
Condition*Time	4, 93.7	0.92	0.45	4, 106	0.29	0.89	4, 102	0.51	0.73
**Trial Day**	1, 64.9	0.1	0.76	1, 68.3	4.08	0.05	1, 46.6	1.02	0.32
**Log(Mass)**	1, 57.7	0.12	0.73	1, 34.9	0.03	0.86	1, 34	0.33	0.57
**Stimulus position**	1, 85.8	1.89	0.17	1, 99	0.001	0.98	1, 93.8	3.68	0.06

Results of 3 models run using (1) ‘Self-grooming’, (2) Rearing and (3) Digging as dependent variables. There was no evidence of a significant interaction between stimulus type and presentation time for these three variables; nor was there a significant main effect of stimulus type on any of these behaviors. Stimulus refers to the type of female stimulus condition presented, time is related to before or after the stimulus presentation. Stimulus position refers to the side of the cage that the stimulus was presented on. Significant values are in bold.

Trial day significantly affected investigative (Table 1) behavior; males decreased the proportion of time they spent investigating the playback stimulus (β = -0.02 ± 0.005) as the experiment progressed; in contrast, animals explored the non-stimulus circle (i.e., where no playback was presented) as the trial day increased (β = 0.00y ± 0.003). Stimulus playback position also significantly affected the amount of investigative time spent at both the playback position and the alternative side (Table 1). Males spent more time at side 1 (0.41 ± 0.02) compared to side 2 (0.27 ± .02) when investigating the playback side. In contrast, males spent more time investigating side 2 (0.34 ± .01) than side 1 (0.20 ± .01) when investigating the alternative side of the arena (i.e., with no playback). This result suggest that our subjects had a side bias which justifies the inclusion of this factor as a covariate in our statistical model. Male mass did not significantly affect the proportion of time spent performing any of the behaviors analyzed (Tables 1 and 2).

### Male vocal behaviors

**Fig 3:**
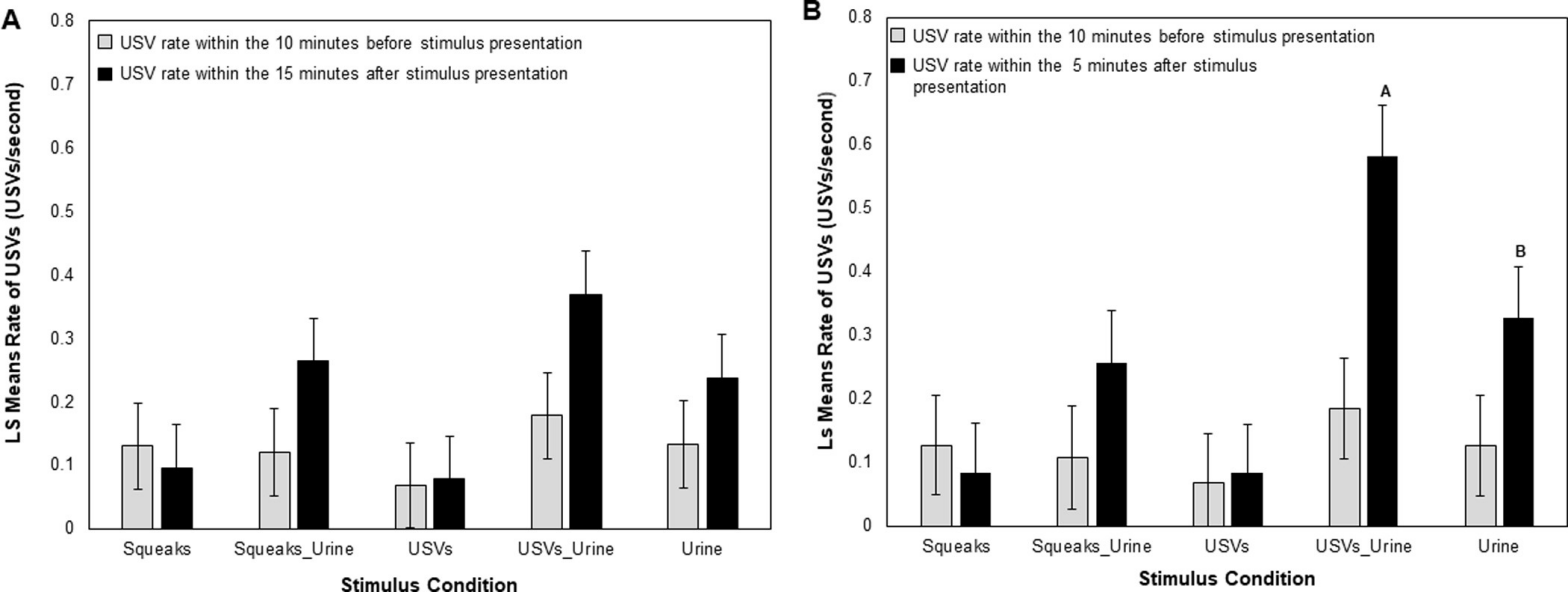
Male total USV rates are affected by stimulus condition. Panel A shows the Least Squares (LS) Means for the total USV (i.e., both harmonic and non-harmonic USVs) rate across 15 minutes after the presentation of the stimulus. Panel B shows the LS Means for the total USV rate across 5 minutes after the stimulus presentation. Gray bars refer to before the stimulus was presented and black bars refer to after stimulus presentation but note the difference in time between panels A and B. Letters denote significant interaction between presentation time (i.e., before and after stimulus presentation) and a given condition. The different letters indicate statistical difference between the USV rates across condition types.

Overall we recorded a total of 18, 682 USVs from our males Here we discuss (1) total USV rates (i.e., the sum of USVs that were both 50 kHz harmonics and ‘other’ USV types) and (2) harmonic USV rates. Males varied considerably in their vocal production, from a range of 123 total vocalizations across all trials to 7,286 total vocalizations across all trials. Interestingly, in a pilot study from 5 males we also found that males do not change their vocal patterns with the presentation of urine collected from females in estrus versus diestrus (F_1,16.4_ = 0.32, P = 0.58; see S1 File). This range of variation has been recorded in previous experiments from our lab [12], and is also a prominent feature of wild-derived populations of *Mus musculus* in the lab [25, 26]. In contrast to non-vocal behaviors, male vocal behaviors were differentially influenced by urine and vocalization presentation. The total USV rate of males both before and after the presentation of the stimuli were initially compared between the first 10 minutes of the trial (i.e., before stimulus presentation) and the final 15 minutes of the trial (i.e., during and after the stimulus presentation). There was no significant interaction between stimulus treatment and stimulus presentation time (see Table 3 and Fig 3A) but there was a significant main effect of stimulus presentation time (Table 3), such that males produce a higher rate of total USVs after the presentation of the stimulus (0.21 ± .05) than before (0.13 ± 0.05).

**Table 3:** Male total USV rate within 5 minutes is dependent on stimulus condition.

	Total USV Rate (5 Mins post presentation)			Harmonic USV Rate (5 Mins post presentation)			Total USV Rate (15 Mins post presentation)		
	D.F.	F value	P value	D.F.	F value	P value	D.F.	F value	P value
**Stimulus**	**4, 24.6**	**5.33**	**0.003**	4,70.7	2.26	0.07	4, 27.9	2.55	0.06
**Time (Before or After)**	**1, 46**	**13.71**	**< 0.001**	1, 39.4	0.21	0.65	**1, 26.9**	**10.85**	**0.002**
Stimulus*Time	**4, 98.8**	**4.08**	**0.004**	4, 98.5	1.98	0.1	4, 68.5	2.44	0.06
**Trial Day**	1, 73.4	0.27	0.61	1, 70.9	0.12	0.73	1, 55.2	0.59	0.45
**Log(Mass)**	1, 53.5	1.05	0.31	1,44	1.49	0.23	1, 52.5	1.49	0.23
**Stimulus position**	1, 87.3	2.43	0.12	1, 76.2	2.68	0.11	1, 61.5	1.44	0.24

Results from three models showing the effect of stimulus condition on (1) Total USV rate for 5 minutes after the stimulus presentation, (2) Harmonic USV rate for 5 minutes after the stimulus presentation and (3) Total USV rate for 15 minutes after the stimulus presentation. Condition indicates the type of stimulus presented whereas time represents before or after the presentation of the stimulus. Stimulus position indicates which side of the cage the stimulus was presented on. Bolded values indicate statistical significance.

**Fig 4:**
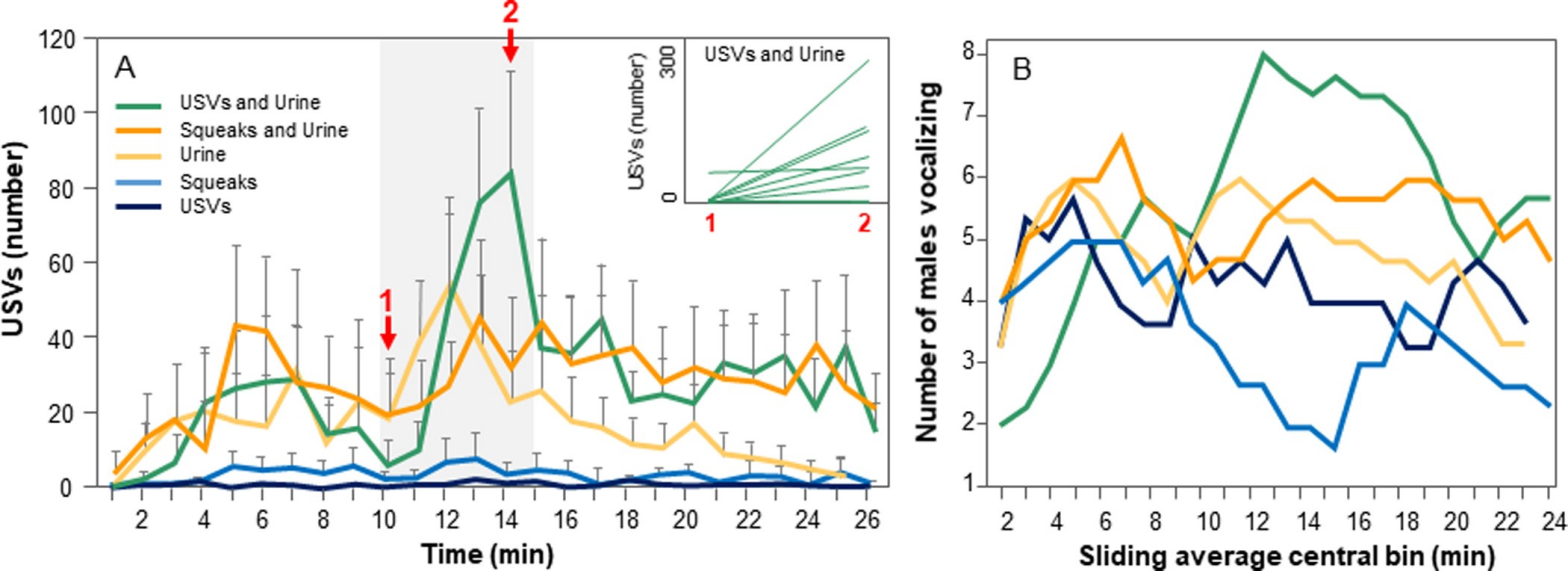
Time-course of male USV production. Panel A shows the mean number of total USVs produced (i.e, both harmonic and non-harmonic USVs) per male in different stimulus treatments. Gray shade = duration of playback. Error bars = S.E.M. *Inset* represents changes in total USV number for individual males between time points 1 and 2 in the urine + USV treatment. Panel B shows the numbers of males vocalizing over the treatments shown in A. Values represent the sliding average of three one-minute bins.

Previous research has established that the rate of male calling substantially decreases after the initial presentation of a female stimulus [15, 58] and that females habituate quickly to the experimental playback of calls [23]. We therefore investigated the time course of male USV production after the stimulus and found that the majority of the USVs were produced within 5 minutes after the beginning of the stimulus presentation (Fig 4A).

When we limited our analysis to the total USV rate within these first five minutes, we found a significant interaction between stimulus presentation and treatment (Table 3 and Fig 3B.) A posthoc analysis reveals that this interaction was driven by treatment differences after the presentation of the stimulus, rather than before (see S3 File and Fig 4A). Interestingly, neither the presentation of squeaks (t_81.6_ = -0.51, p = 0.71) nor USVs (t_81.6_ = 0.19, p = 0.91) in isolation changed the total rate of USV production (Fig 3B). After the presentation of USVs paired with urine, males responded with a great increase in their total USV rate (t_92.2_ = 4.58, P < 0.001). This increase was significantly greater than their response to urine alone (t_64.7_ = 2.62, p = 0.03) (Fig 3B). The addition of urine to squeaks, however, did not change the proportion of USVs given before or after the stimulus (t_93.3_ = 1.66, p = 0.14) (Fig 3B).

Fig 4A illustrates the mean numbers of male USVs produced over time in one-minute-bins, with the time course of the playback of vocalizations indicated by the shaded area. Urine was present from the start of the shaded area through the duration of the experiment. In this figure, the numbers of males vocalizing increases in the time bins following the combined presentation of urine and USVs. Interestingly, the number of vocalizing males declines during the presentation of squeaks alone (Fig 4B), although the average rate of squeak production does not decrease (Fig 4A). The transient increase in the male call rate following the USV and urine presentation (green line) is clearly visible in this plot. In Fig 4A it also becomes apparent that while there were no statistical differences between treatments before the presentation of urine stimuli (S3 File), there is a non-significant trend for males to vocalize more during a trial that involves olfactory stimuli. It is thus possible that despite our efforts to eliminate odor cues (i.e., by always using new tubing), males may have been able to detect urine traces prior to their presentation. Nevertheless, we do see a significant effect of the urine presentation. Furthermore, because we always compared the USV rate from before to after we are confident in our statistical interpretation. In addition, although there was variation among males in their overall call rate, seven of nine males changed their USVs in the same direction following USV and urine presentation (Fig 4A, inset). It is also interesting to note that although male vocalizations declined to low levels following the presentation of urine alone, vocalization rates for urine plus either USVs or squeaks remained somewhat elevated, even after termination of the playback. Thus, a distinguishing feature of the male vocal responses to female stimuli may include the time course as well as vocalization rate. An effect of stimulus presentation on male vocalization is also reflected in the numbers of males calling over time. This is depicted in Fig 4B, which is a sliding window average of the numbers of males vocalizing in three one-minute time bins. The increase in the number of males vocalizing following the presentation of urine+ USVs (green line) reinforces the stimulatory effect of this treatment. Additionally, this plot reveals a decline in the number of vocalizing males following the presentation of BBVs alone (light blue line).

In addition to our analysis of total USV rate, we also examined whether males altered their use of 50 kHz-harmonic USVs which our lab and others have previously documented as being associated with male mounting behaviors in an intersexual interaction [12, 13, 50, 51]. The 50 kHz harmonic USV contains portions with two distinct harmonically related frequency bands, with a fundamental frequency near 50 kHz. We also measured the duration and the dominant frequency (i.e., frequency at maximum intensity) as a function of stimulus type or presentation time. We found no evidence to suggest that stimulus type affected the rate of 50 kHz harmonic USVs (Table 3). There was no significant interaction between stimulus type and presentation time, nor were there any main effects of stimulus type or presentation time (Table 3). Overall, males gave relatively few 50 kHz harmonic calls: of the 11, 954 total USVs given in the 5 minutes following playback, only 1, 425 (roughly 12% of these vocalizations) contained a 50 kHz harmonic. We also did not find evidence to suggest that males altered either the dominant frequency or the duration of their vocalizations with stimulus type (Table 4), but we did find a main effect of presentation time on total duration (Table 4). Males produced longer USVs in the 5 minutes following the playback (0.014 ± 0.001) compared to USVs given before the playback (0.012 ± 0.001). None of the covariates we investigated (i.e., trial day, male mass, or playback side) significantly influenced either the rate of USV production (Table 3) or the spectral or temporal nature of the USVs (Table 4).

**Table 4:** Male USV duration is influenced by the presence of female stimuli.

	Total USV Duration			Total USV Dominant Frequency		
	D.F.	F value	P value	D.F.	F value	P value
**Stimulus**	4, 25.1	1.52	0.23	4, 75.1	0.71	0.59
**Time (Before or After)**	**1, 51.3**	**5.76**	**0.02**	1, 46.7	0.01	0.91
Stimulus*Time	4, 74.3	0.40	0.80	4, 79.8	0.93	0.45
**Trial Day**	1, 71.8	0.02	0.89	1, 63.2	1.09	0.30
**Log(Mass)**	1, 26.6	1.27	0.27	1, 25.6	0.02	0.88
**Stimulus position**	1, 96.3	0.02	0.88	1, 88.5	1.28	0.26

Results from two models showing the effect of stimulus condition and presentation time on total USV duration and USV dominant frequency. Condition indicates the type of stimulus presented whereas time represents before or after the presentation of the stimulus. Stimulus position indicates which side of the cage the stimulus was presented on. Bolded values indicate statistical significance.

## Discussion

The two main findings of this study were that male mice respond to the presence of female signals, and that the specific sensory mode informs male behavior. Males adjusted their investigative behavior depending on whether information was conveyed through auditory or olfactory channels and altered the rate of their vocal courtship depending on whether female squeaks or female USVs were paired with female urine. Specifically, we found a significant interaction between presentation time (i.e., before or after stimulus presentation) and stimulus type for both male investigation and total USV production (i.e., USVs included both harmonic and non-harmonic calls). In our study males did not increase investigation time or USV production to female vocalizations (i.e., squeaks and USVs) presented without urine. In contrast, in conditions where urine was presented without vocalizations, males responded with increased investigation. This is similar to what is seen in female cowbirds evaluating a male’s audiovisual courtship display; females will give a copulatory posture to just the male’s song without the visual component [59] but will not give such a posture to the visual wingspread in isolation [60]. The multiple messages hypothesis predicts that if a behavioral response to each signal component is different, the information contained in each mode is also different. As we predicted, our data upholds the multiple messages hypothesis of non-redundant signals, as males responded differentially to the presence of each signal component in isolation.

We also found that when female vocalizations were paired with urine, males modified their vocal output depending on the specific female vocalization (e.g., squeaks or USVS) they were presented with. Overall, we also found a fairly high degree of individual variation in the vocal production of the males included in our study; this individual variation was somewhat unsurprising as previous work in our lab [12] and others [25, 26] have also reported substantial individual differences. Our experimental design controlled for this in part by (1) using a comparison of before and after stimulus presentation to look in the proportion of change in vocal production rather than the overall value and (2) using a repeated measures design. The use of this design, however, limited our ability to test across a relatively large number of individuals which could affect the generalizability of our results. Nevertheless, we are confident in our overall findings and see robust patterns in our data. For example, we found that in our USV and urine condition (i.e., where we predicted the highest degree of vocal production) that the majority of males responded with increased USVs and that this condition also had the highest number of males vocalizing. We discuss the future implications of these findings, including how the presence of female chemical cues provides the social context for male listeners, how this changes our understanding of simultaneous versus sequential multimodal signals, and how we determine the function of a multimodal signal.

### Olfactory stimuli provide context for female vocalizations

Male USV production was the highest when males were presented with the combination of female USVs and female urine. In contrast, when males were presented with the combination of female urine and squeaks, males did not alter their USV production from before the stimulus to after the stimulus. These findings support our predictions that male USV production should be the highest in response to the combination of female USVs and urine. Additionally, we also found that there was no difference between the stimuli that produced the lowest amount of USVs: female squeaks, female USVs, and the combination of female squeaks and urine. These results uphold the hypothesis that female squeaks and female USVs have two different functions in intersexual communication. Squeaks may signal female rejection as these vocalizations are often paired with other, negatively-valenced behaviors like kicking and lunging [13, 35, 61]. As a result, males may reduce their vocalization rate. This result corroborates previous findings demonstrating that males are less likely to mount females that produce many squeaks at the beginning of an interaction [13]. In contrast, female USVs, when paired with female urine, may convey female receptivity, especially when they temporally overlap with those of males [32].

In contrast to what we had originally predicted, males did not produce more USVs in response to female USVs alone. In fact, female vocalizations alone (squeaks or USVs) did not produce any changes in male vocalization efforts in the before versus after conditions. Nevertheless, relative to urine alone, female USVs significantly increase male USVs whereas female squeaks do not. Together these findings suggest that the olfactory cue sets the context for the female vocalization [6].The context hypothesis posits that one signal component provides the context in which a receiver can interpret a secondary signal component [6]. The ability of one signal component to affect the response of another is one example of how signal components can interact [6]. Importantly, the context-hypothesis and the multiple messages hypothesis are not mutually exclusive [6]. In fact, interactions between modes are most likely to occur when the signal components are non-redundant [6, 7, 9, 62]. The context hypothesis has also been supported in snapping shrimp (*Alpheus heterochaelis*) males, which show a greater behavioral response to the visual display of an open chela depending on whether the chemical signal it is paired with originated from a male or female (Hughes 1996).

Male mice can unequivocally differentiate between female and male urine [18], but there is mixed evidence on whether males and females produce different USVs [33, 63–66] and even less is known about whether males and females produce different squeaks. Within a single sex we know that female squeaks elicited during an intersexual encounter are similar to those elicited during restraint [34] but there is still a lack of understanding about male squeaks and propensity. In studying sex-differences in USV production, Hammerschmidt et al. 2012 found substantial structural and functional overlap between female USVs and male USVs, but their experimental paradigm examined vocalizations produced in an intrasexual context [33]. A more recent experiment found significant sex differences in frequency bandwidth and frequency change over time for USVs, but there was still much overlap in these two parameters between sexes [65]. In addition to spectral and temporal measurements, new evidence from C57BL/6 J mice suggests that males and females use different types of calls depending on the social context [51]. Males were found to use primarily ‘simple’ USVs when paired with other males, while females used more ‘complex’ calls when paired with other females; intersexual recordings of males and females showed a combination of both call types [51]. Harmonic calls, in contrast, were given only during reproductive (e.g., mounting) behaviors [51]. This use of harmonic call structure is similar to what we have previously observed [12, 13] and also collaborates our current findings. We did not observe a difference in the rate of harmonic USVs between any of our stimulus conditions, perhaps because these USVs are very explicitly only given during mounting behaviors.

To the best of our knowledge, no studies have tested whether mice can behaviorally discriminate the differences between male and female USVs, or male and female squeaks. Thus, in the current study, males presented with female vocalizations without urine may have interpreted the vocalizations as another conspecific male rather than as a female. Resident males tend to produce fewer vocalizations to a male intruder than a female intruder [33]. The addition of female urine to female USVs may have provided context to the males and resulted in an increase in USV production. Male chemical signals added to male USVs also alter female preference for male USVs [27]. Females in diestrus discriminate between the USVs of males from different strains, but only when they were first presented with chemical cues from these males [27]. Similarly, Grimsley et al. (2013) found that male mice avoided female squeaks when they were paired with a predatory odor but were relatively more attracted to the vocalization when they were paired with female urine or a neutral stimulus (Grimsley et al. 2013). Interestingly, these findings mapped onto the neuronal responses in the basolateral amygdala, a brain region known for its role in shaping the salience of sensory stimuli (Grimsley et al. 2013).

In the current study we used pooled female urine from across females presumably in different phases of the estrous cycle. In regularly cycling rodents, females go through the estrous stages roughly every 4–5 days [45] and this is what our lab has demonstrated previously ([12, 13, 42, 43]. In a pilot study we found that males gave the same rate of total USVs across both estrus-pooled urine and diestrus-pooled urine (see S1 File) suggesting that males respond similarly across these conditions. This also supports previous findings in our lab [12].

We collected urine twice daily from animals over the course of two months to generate a pool that included both diestrus and estrous urine. Similar to other strains like male C57Bl/6 and male BTBR T+ tf [67], the strain used in our lab (i.e., CBA/J), do not change the number or rate of USVs produced with female reproductive state [12] (but see [68]). Rather, male CBA/Js produce USVs of longer duration, greater bandwidth and higher frequency when engaging with estrous versus diestrus females. Furthermore, male USV duration has also been found to be influenced by the presence or absence of live female partners [12]. Female removal from a male-female interaction led to a shortening of USVs [12]. Interestingly, this corresponds well to our current findings in which we found an increase in USV duration with the addition of female stimuli. Taken together, our results demonstrate that non-auditory, chemical cues may provide the context to by which ambiguous sounds can be distinguished.

### Context provided by differences in active space between signaling components

Olfactory and acoustic signals vary substantially in their active spaces (i.e., the volume of a medium, or a period of time, within which a receiver can detect a signal) [69]. Deposited scent marks like those in urine can remain in the environments and can therefore be detected over longer timescales while acoustic signals are more transient. This difference between chemical scent marks and acoustic signals allows for separation in terms of how and when the receiver processes these signal components. This ‘separability’ of signal components has also been described as components that are ‘free’ [9, 70] and the signal design is inherently different from components that are more ‘fixed.” A fixed multimodal signal, like that of the required movement of human lips with the production of sounds, has greater potential to be evaluated simultaneously by the receiver. Whether or not there is an intrinsic difference in multimodal signal components that are evaluated simultaneously or sequentially remains an important but rather understudied question in multimodal communication [71, 72], including specifically within mice [20].

When signals do not share a temporal or spatial active space, the signaling modality that has the largest active space may provide behavioral context for the signal with the smaller active space. Animals are more likely to first encounter the signaling component of a modality that has a larger active space; signal components with larger active space therefore have greater potential to provide context for other subsequent modalities. In support of this, previous work in guppies (*Poecilia reticulata*), a visually-dominated species, found that the presence of an olfactory conspecific alarm cue presented before an ambiguous water disturbance event modulated the fishes subsequent responses [73]. In the current experiment we presented olfactory and acoustic stimuli to the males at the same time but the olfactory stimulus remained in the environment after the playback of the vocalizations ended. This design was intentional as it captures the reality of an animal encountering olfactory and acoustic signal components. Nevertheless, it would be worth systematically exploring whether a receiver's behavior differs with the changes in temporal and spatial overlap of multimodal signal components. Interestingly, research in túngara frogs (*Engystomops pustulosus*) shows that placing a visual stimulus of a calling frog in between the playback of two auditory components (e.g., the frog’s distinctive ‘whine’ and ‘chuck’) can perceptually “rescue” this non-natural display to make it just as attractive as a temporally synchronous visual and acoustic display [74]. Such ‘rescuing’ may be beneficial in uncertain environments where signaling components in different modalities may have differential environmental pressures shaping the transmission of the signals. For example, olfactory components may be subject to environmental perturbations like wind; it would be interesting to examine the effects of temporal displacement between olfactory and auditory cues on receiver processing.

### Multimodal signal classification dependent on behavior analyzed

The temporal difference between auditory and olfactory signals may explain one of the more intriguing results of our current study: male investigative behavior demonstrates that chemical information is *dominant* to acoustic information, while male USV production shows that chemical information instead *modulates* acoustic information [8, 9]. We predicted that male investigative behaviors would be the lowest when males were presented with the combination of squeaks and urine, and the highest when males were presented with urine and USVs. Contrary to this, we found that male investigative behavior was high if urine was present, and statistically indistinguishable whether it was presented with squeaks, USVs, or alone. Dominance of a sensory modality is relatively rare in the multimodal literature [9, 75] and is predicted to occur in scenarios when the cost of acquiring more information is high [76].

A notable example is the dominance of visual information over auditory cues in adult humans [77]. This phenomenon has been called the Colavita effect; human subjects presented with an auditory tone and a simultaneous flashing light often report seeing the light first [78]. This effect is so robust that it is reported even when the auditory tone is presented at twice the subjective intensity of the light [78]. Dominance has also been shown several times in the *Schizocosa* wolf spiders [75, 79]. In several of these species, elaborate leg ornamentation does not affect the degree of mating success; rather, mating success is predicted by the presence of seismic signal. The evolution of elaborate visual displays in these species are therefore predicted to function to increase male detectability in an environment where seismic signals may not propagate far [75, 80]. In mice, female urine may be the first cue of a nearby female to a potential mate but does not necessarily reliably indicate the presence of a female at that moment in time. Investigative behaviors, therefore, may facilitate the acquisition of more information about the presence of a potential mate.

In our models of investigation, it is also important to note that several covariates that we included in our model were statistically significantly: namely both trial day and stimulus position (i.e., which side of the cage the stimulus was presented on). We included trial day as a factor because of our repeated measures design and previous findings that mice often habituate to the testing environment [23]. Indeed, we did find evidence of habituation in our mice: males spent less time investigating the stimulus circle or more time investigating the non-stimulus circle as the experiment progressed from Day 1 to Day 10. In addition, males also appeared to have a side bias and preferred to spend more time investigating the stimulus circle when it was on position 1 rather than position 2. We attempted to control for this side bias by randomizing which side the stimulus was presented in our experimental design. Nevertheless, including both of these factors as covariates in our model allows us to still explore the effects of stimulus treatment while controlling for outside factors (i.e., trial day and stimulus position). Our results unequivocally show that male investigative behaviors are dominantly influenced by the addition of chemical signals. In contrast to the dominance of chemical signals in investigation, we found that the addition of female vocalizations to female urine modulated male vocal production. Modulation occurs when one signal component modulates (i.e., in our results, increased) the response to an additional sensory component. In comparison to dominance, modulation is the most common example of non-redundant signal components [9] and has been confirmed in numerous other species including birds [62], frogs [81], and honey bees [82]. In mice, the presence of an acoustic signal is a reliable indicator of a nearby, broadcasting conspecific. When this information is paired within the context of female urine, the predation risk from vocalizing could potentially be substantially reduced.

Our results demonstrate the importance of evaluating multiple components of the receiver's behavior to determine the function of multimodal signal components [9]. Just as signals are comprised of multiple components, responses to these signals may be multifaceted and complex [83, 84] and influenced by the environment the signals are transmitted through. This is not necessarily a new idea to the multimodal communication literature [9] but it is relatively rare to find studies that use multiple estimates of receiver behavior under different environmental conditions [85]. Such a systems approach to multimodal signaling will benefit the ability of researchers to examine evolutionary selection on signals across different environments [84].

## Conclusions

This study systemically describes the function of chemical and acoustic signaling components in the house mouse. We found that males respond to female multimodal signaling components and these components contained different information supporting the multiple messages hypothesis. Moreover, the presence of female urine provided social context for the male who subsequently adjusted his courtship vocalization effort. Thus, our study provides evidence that males, although typically thought of as senders of communication signals, can adjust their courtship behaviors depending on the female signals. An interesting next step would be to examine whether similar or different patterns emerge within a intrasexual context between pairs of males or pairs of females as mice are highly social and therefore spend much time communicating with both sexes. A recent study of diurnal geckos (*Cnemaspis mysoruensis*), for example, found that while male chemical secretions alone were sufficient for females to respond, males required both male visual and chemical secretions to be present [86]. Studies that compare both the responses to male and female multimodal cues across the sexes are necessary but lacking in the field of animal communication. In sum, mice are becoming increasingly used as models to address basic questions in communication and our results illustrate the complex and dynamic nature of mouse social interactions.

## Supporting information

S1 File Male total USV production not influenced by female estrous state. Urine was collected daily from 23 additional females and their estrous state assessed and classified between estrous and diestrous. Urine was presented in a pilot study to 5 additional males to determine whether USV rates changed depending on the urine presented. Each male was presented with each urine type in a repeated measures study. We found no evidence to suggest that urine type influences the rate of male vocalizations.
(DOCX)

S2 File Description of the experimental paradigm for each male. This table shows the experimental condition that each male (males 1–9) underwent on each trial day (days 1–10). There are 5 different stimulus conditions: USVs, Squeaks, Urine, USVs and Urine, and Squeaks and Urine. Each male was exposed to each condition randomly and with one replacement such that each male was exposed to each of the stimulus conditions twice. This repeated measures design allowed us to separate the differences we see between males to each stimulus from the variation within each individual male.
(PDF)

S3 File Results of a posthoc analysis shows that the rate of USVs prior to stimlus presentation did not differ between trial types. Here we show the results of a posthoc analysis comparing the interaction between stimulus type and presentation time (i.e., before or after the presentation of the stimulus). We find no evidence to suggest that any of the stimulus conditions varied in their rates of total USV production between treatment groups before the presntation of the stimulus.
(DOCX)
