# Supplementary material for: Male mice adjust courtship behavior in response to female multimodal signals
Source: PLoS One. 2020 Apr 2;15(4):e0229302. doi: 10.1371/journal.pone.0229302 (PMC7117945; doi:10.1371/journal.pone.0229302)
Supplement: S1 File — Urine was collected daily from 23 additional females and their estrous state assessed and classified between estrous and diestrous. Urine was presented in a pilot study to 5 additional males to determine whether USV rates changed depending on the urine presented. Each male was presented with each urine type in a repeated measures study. We found no evidence to suggest that urine type influences the rate of male vocalizations. (DOCX) [file pone.0229302.s001.docx]

Supplementary Text File 1

An additional 28 animals were used in this pilot study: 23 females were used to collect estrous phase-specific urine and 5 additional males were used to assess the effect of female estrous state on vocal output of males. All females were housed within same-sex groups in pairs or groups of three; the males were housed in one pair and one group of three. All animals were given ad-libitum access to food and water.

Urine was collected daily from 23 female mice and stored in individual tubes over the course of one month. As described in the methods in the main text, urine was collected by handling a mouse over a clean sheet of aluminum foil. After urination, the urine was immediately pipetted into a centrifuge tube on dry ice. The date and female identity were recorded for each individual tube and they were stored in a -80 freezer.

After urine collection each female was then assessed to determine her estrous state. Vaginal lavage with 50 µL of sterile saline was used to create a smear on a microscope slide. Slides were then collectively stained with Giemsa and then analyzed under a microscope following previous work in our lab (1-4). Estrous phases were determined by the relative number of different cell types within an individual. The presence of leukocytes indicated diestrus and the presence of only cornified epithelial cells indicated estrus. Once estrous state for each female across each day was determined, urine across these 23 females was pooled into either—estrous versus diestrous urine pools.

We then completed a pilot study to assess how urine from females in estrus versus diestrus affected the vocal output of male mice. 5 adult males (aged 9-10 weeks) with previous sexual experience were exposed to both diestrus and estrus urine in a repeated measures design in which each male was exposed to each condition (estrous versus diestrous urine) twice. On a given trial day, males were placed in the experimental arena as described in the methods section and, after 10 minutes of habituation, were presented randomly either with pooled diestrous urine or pooled estrous urine. Recording continued for an additional 15 minutes after urine presentation. Trials lasted for 4 days with each male undergoing on trial per day for a total of 4 total trials, with two total presentations of each urine type. During one trial it was discovered that the urine was not presented to the animal as expected so we removed this trial from the analysis so that one male only underwent 3 total trials.

Avisoft SASLab Pro software (Avisoft Bioacoustics) was used to analyze all spectrogram recordings of USVs that the males produced during trials. We high-pass filtered all audio files above 35 kHz to remove the majority of the background noise and then determined the rate of USV production across all trials.

A repeated measures ANOVA running Proc Mixed in SAS (Version 9.3) was used to assess differences between vocal rates in males exposed to both diestrus and estrus urine. Similar to the analyses explained in the full text, we used male identity as a repeated factor to investigate the statistical interaction between playback time (i.e., before or after the urine was presented) and the treatment (estrus versus diestrus urine). We also included trial day and the mass (log transformed) of the males as a covariate in the analysis. Our dependent variable (i.e., Total USV rate) was arcsin transformed to meet the normality assumption and male and stimulus treatment within male were both included as random statements. In addition, we specified an autoregressive covariance structure and the Kenward-Rogers method was used to calculate the degrees of freedom.

We found no evidence of a statistical interaction between estrus state and before versus after the urine was presented. This indicates that males do not change their USV production in different ways depending on the estrus phase of the female (F_1,16.4_ = 0.32, P = 0.58). Moreover, there was also no main effect of urine type (F_1,4.44_ = 0.02, P = 0.90) on male USV production. As expected, the time of stimulus presentation did show a main effect such that males produced USVs at a higher rate after the urine was presented (F_1,5.14_ = 7.12, P = 0.04). Neither trial day (F_1,13.3_ = 0.01, P = 0.91) nor mass of the male (F_1,5.88_ = 0.41, P = 0.55) affected USV production. Taken together, this evidence suggests that overall male vocal production does not differ depending on whether the female was in estrus or diestrus when urine was collected. This result is not necessarily all together surprising as previous work in our lab has shown that male calling rate does not change with female estrous state (3)

Figure caption: **Total USV rate does not change depending on estrous state**. Males (5) were presented with both estrus and diestrus urine and their vocal behavior was assessed both before the urine presentation and after. We found no evidence of an interaction between presentation time (i.e., before or after urine presentation) and urine type; nor did we find a significant main effect of urine type. Presentation time did have a significant main effect such that after the urine was presented, a greater rate of USVs occurred.

1. Hanson JL, Hurley LM. Serotonin, estrus, and social context influence c-fos immunoreactivity in the inferior colliculus. Behav Neurosci. 2016;130(6):600-13.

2. Hanson JL, Hurley LM. Context-dependent fluctuation of serotonin in the auditory midbrain: the influence of sex, reproductive state and experience. J Exp Biol. 2014;217(4):526-35.

3. Hanson JL, Hurley LM. Female Presence and Estrous State Influence Mouse Ultrasonic Courtship Vocalizations. Plos One. 2012;7(7).

4. Goldman JM, Murr AS, Cooper RL. The rodent estrous cycle: Characterization of vaginal cytology and its utility in toxicological studies. Birth Defects Res B. 2007;80(2):84-97.
