# Supplementary material for: Male mice adjust courtship behavior in response to female multimodal signals
Source: PLoS One. 2020 Apr 2;15(4):e0229302. doi: 10.1371/journal.pone.0229302 (PMC7117945; doi:10.1371/journal.pone.0229302)
Supplement: S2 File — This table shows the experimental condition that each male (males 1–9) underwent on each trial day (days 1–10). There are 5 different stimulus conditions: USVs, Squeaks, Urine, USVs and Urine, and Squeaks and Urine. Each male was exposed to each condition randomly and with one replacement such that each male was exposed to each of the stimulus conditions twice. This repeated measures design allowed us to separate the differences we see between males to each stimulus from the variation within each individual male. (PDF) [file pone.0229302.s002.pdf]

		INDIVIDUAL MALE								
		Male 1	Male 2	Male 3	Male 4	Male 5	Male 6	Male 7	Male 8	Male 9
TRIAL DAY	Day 1	USVs and Urine	USVs	Squeaks and Urine	Squeaks and Urine	USVs and Urine	Urine	Urine	Squeaks and Urine	Squeaks and Urine
	Day 2	Squeaks	Squeaks and Urine	USVs and Urine	USVs and Urine	Squeaks	Squeaks and Urine	USVs and Urine	USVs	USVs and Urine
	Day 3	Urine	USVs	Squeaks	Squeaks	USVSs and Urine	Urine	Urine	USVs and Urine	USVs and Urine
	Day 4	Squeaks and Urine	Squeaks and Urine	Urine	USVs and Urine	Squeaks and Urine	USVs	Squeaks	USVs	Urine
	Day 5	Urine	USVs and Urine	USVs	Squeaks and Urine	Urine	Squeaks	USVs and Urine	Squeaks	Squeaks and Urine
	Day 6	USVs	Squeaks	Squeaks	USVSs	Urine	Squeaks and Urine	Squeaks	Squeaks	USVs
	Day 7	Squeaks	Squeaks	USVs	Squeaks	USVs	USVs and Urine	Squeaks and Urine	Urine	USVs
	Day 8	USVs	Urine	Squeaks and Urine	Urine	Squeaks and Urine	USVs	USVs	USVs and Urine	Urine
	Day 9	Squeaks and Urine	Urine	Urine	USVs	Squeaks	Squeaks	USVs	Urine	Squeaks
	Day 10	USVs and Urine	USVs and Urine	USVs and Urine	Urine	USVs	USVs and Urine	Squeaks and Urine	Squeaks and Urine	Squeaks
