# Supplementary material for: Male mice adjust courtship behavior in response to female multimodal signals
Source: PLoS One. 2020 Apr 2;15(4):e0229302. doi: 10.1371/journal.pone.0229302 (PMC7117945; doi:10.1371/journal.pone.0229302)
Supplement: S3 File — Here we show the results of a posthoc analysis comparing the interaction between stimulus type and presentation time (i.e., before or after the presentation of the stimulus). We find no evidence to suggest that any of the stimulus conditions varied in their rates of total USV production between treatment groups before the presntation of the stimulus. (DOCX) [file pone.0229302.s003.docx]

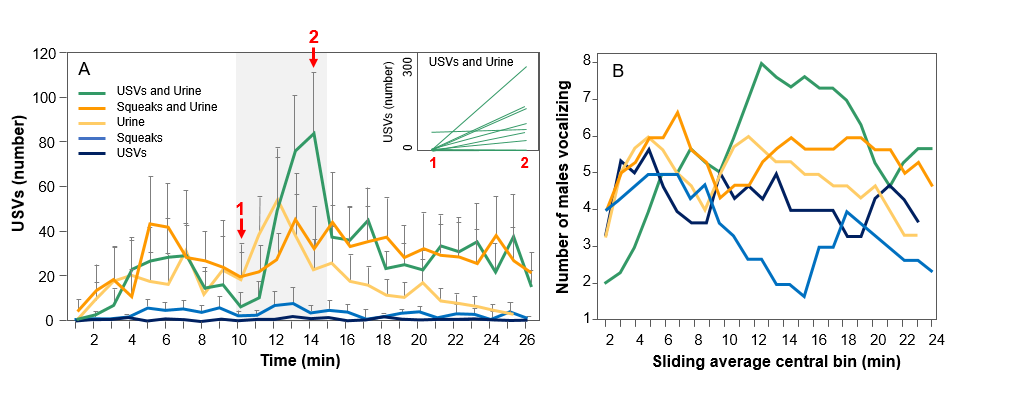


The graph above shows the average production of USVs for all trials. Lines are constructed by averaging USV counts across all males. While there may appear to be differences in the means across treatment groups prior to the stimulus being presented (i.e., Bin 20), this difference is not statistically significant. Below we have presented the posthoc comparisons between each treatment group prior to the stimulus presentation; no comparison is significantly different. In designing the study, we incorporated foreknowledge of the variation in male vocal behavior. Past studies in our lab have shown that almost all males vocalize when placed with females, but some males vocalize copiously, and some do not. This kind of between-male variation in call rate is also a prominent feature of wild-derived populations of *Mus musculus* in the lab (Musolf, Hoffmann et al. 2010, von Merten, Hoier et al. 2014).

**Squeaks and Squeaks_Urine, (t = 0.20 ± 0.1, P = 0.84)**

**Squeaks and USVs, (t =0.64 ± 0.09, P = 0.53)**

**Squeaks and USVs_Urine, (t =-0.61 ± 0.09, P = 0.54)**

**Squeaks and Urine, (t =0.01 ± 0.09, P = 0.99)**

**Squeaks_Urine and USVs, (t = 0.42 ± 0.10, P= 0.68)**

**Squeaks_Urine and USVs_Urine , (t = -0.79 ± 0.10, P = 0.43)**

**Squeaks_Urine and Urine, (t = -0.19 ± 0.10, P = 0.85)**

**USVs and USVs_Urine, (t = -1.25 ± 0.09, P = 0.22)**

**USVs and Urine, (t = -0.62 ± 0.09, P = 0.53)**

**USVs_Urine and Urine, (t = 0.62 ± 0.09, P = 0.54)**
